# A weather features dataset for prediction of short-term rainfall quantities in Uganda

**DOI:** 10.1016/j.dib.2023.109613

**Published:** 2023-09-25

**Authors:** Andrew Gahwera Tumusiime, Odongo Steven Eyobu, Isaac Mugume, Tonny J. Oyana

**Affiliations:** a College of Computing and IS, Makerere University, P.O Box, 7062, Kampala, Uganda; b College of Agricultural and Environmental Sciences, Makerere University, P.O Box, 7062, Kampala, Uganda

**Keywords:** Climate R-package, Python, Dataset, Meteorology, Short-term rainfall prediction, Deep learning

## Abstract

Weather data is of great importance to the development of weather prediction models. However, the availability and quality of this data remains a significant challenge for most researchers around the world. In Uganda, obtaining observational weather data is very challenging due to the sparse distribution of weather stations and inconsistent data records. This has created critical gaps in data availability to run and develop efficient weather prediction models. To bridge this gap, we obtained country-specific time series hourly observational weather data. The data period is from 2020 to 2022 of 11 weather stations distributed in the four regions of Uganda. The data was accessed from the Ogimet data repository using the “climate” R-package. The automated procedures in the R-programming language environment allowed us to download user-defined data at a time resolution from an hourly to an annual basis. However, the raw data acquired cannot be used to learn rainfall patterns because it includes duplicates and non-uniform data. Therefore, this article presents a prepared and cleaned dataset that can be used for the prediction of short-term rainfall quantities in Uganda.

Specifications Table

**Table:** 

Subject	Weather Prediction, Deep Learning
Specific subject area	Short-term Rainfall Prediction
Data format	Raw and analysed data
Type of data	Tables and Figures
Description of data collection	The ogimet data repository was used to obtain the observational weather data. Date, air temperature at 2 m above ground level (TC), dew point temperature at 2 m above ground level (TdC), relative humidity (Hr), wind direction (ddd), wind speed (ffkmh), air pressure at the station's elevation (hPa), precipitation (Precmm), total cloud cover (Nt), cloud cover by high-level cloud fraction (Nh), the height of cloud base (HKm), and visibility (Viskm) were all recorded hourly. We followed the instructions in article [1]. The following were the guidelines: (i) Installing R-studio 4 and higher in the R-programming language environment. (ii) Get the climate R-package at https://github.com/bczernecki. (iii) In the same article, the codes for downloading raw country-specific meteorological data were defined. (iv) The ogimet data repository only provided access to 11 weather stations in Uganda. (v) Each gauge station dataset was saved as a (.csv) file. Following that, the raw data was processed in the Python programming language environment. We cleaned the datasets during data preparation to remove noisy and missing data. The data was normalized using StandardScaler in Sklearn.Pre-processing library in Python [2].
Data source location	The data for the weather gauge stations at Kampala, Entebbe Airport, Jinja, Tororo, Soroti, Gulu, Arua, Masindi, Kasese, Mbarara, and Kabale in Uganda were collected from the Ogimet data repository. Figure 1 displays the location of stations. Table 1 displays the geographical coordinates of the gauge stations, such as latitude and longitude.
Data accessibility	Repository name: Harvard Dataverse Data identification number: doi:10.7910/DVN/PQLYHP Direct URL to data: https://dataverse.harvard.edu/dataset.xhtml?persistentId=doi%3A10.7910%2FDVN%2FPQLYHP

## 1 Value of the Data

• The data in this article can be used to achieve the following: a) improve the prediction of short-term rainfall quantities in Uganda, b) ascertain rainfall patterns in selected Ugandan regions, and c) reduce the uncertainty of rainfall forecast.
• The dataset will help specialists and researchers in meteorology, agriculture, and water resource management predict short-term weather. The cleaned weather data can be used as input to prediction and statistical models. For example, this data can help reduce model underfitting and overfitting.
• This dataset can be utilized by other researcher communities to predict short term rainfall quantities for countries with similar or comparable weather patterns like Uganda. In order to achieve this, comparison of data for the specified weather parameters in our dataset can be made with those of the other countries. Where the comparison of the data is close to similar, it is possible to predict rainfall quantities using the developed prediction model based on our dataset.

## 2 Objective

The objective of this paper article was to provide a dataset of weather information that can be used to aid the development of intelligent rainfall prediction models. This effort seeks to contribute to meteorology, agriculture, and disaster management by giving access to meteorological data for estimating short-term rainfall quantities in Uganda.

## 3 Data Description

**Fig. 1:**
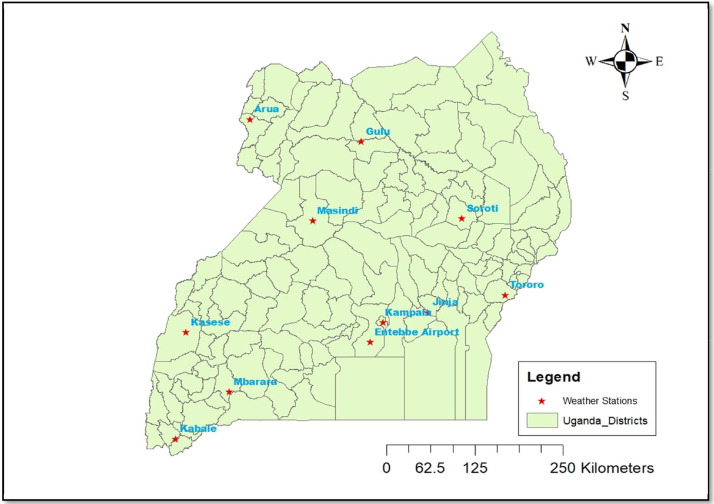
Location of weather stations in Uganda.

**Fig. 2:**
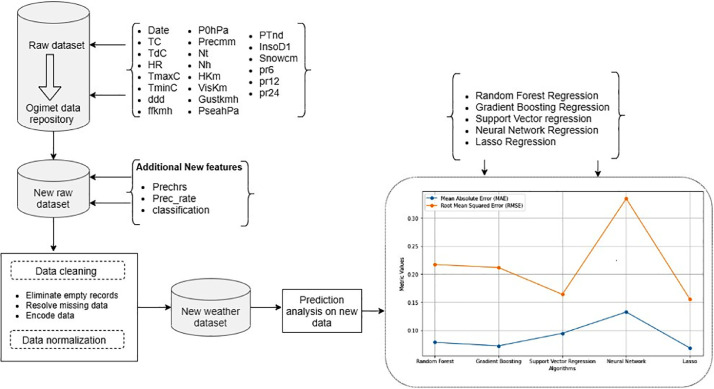
An illustration showing the materials and methods used to prepare the data.

The data presented in this article are observational weather data obtained from eleven (11) weather stations for the different climatology zones of Uganda. Each station had its.csv file at a resolution of hourly to an annual basis. The downloaded data files had approximately 23 features but only 12 features were considered for this experiment because the rest of the features had no data. The features that were considered included: *Date, air temperature at 2 m above ground level (TC), dew point temperature at 2 m above ground level (TdC), relative humidity (Hr), wind direction (dd), wind speed (ffkmh), air pressure at an elevation of the station (hPa), Precipitation (Precmm), total cloud cover (Nt), cloud cover by high-level cloud fraction (Nh), the height of cloud base (HKm), and visibility (Viskm)*. The basic information on the gauge stations, data files, and variables are given in Table 1 and Table 2 respectively. Tables 3 and 4 show the uncleaned and cleaned weather dataset for the Entebbe Airport weather station. Fig. 1 indicates the location map of the meteorological weather stations of Uganda and Fig. 2 the materials and methods used to process the data.

**Table 1:** The information on gauge stations where secondary data was obtained. WMO ID: World Meteorological Organization Identifier of weather stations in Uganda.

WMO ID	Station names	Longitude	Latitude	Altitude
63602	Arua	30.91669	3.050001	1204
63630	Gulu	32.33334	2.750015	1104
63654	Masindi	31.71668	1.683347	1146
63674	Kasese	30.10000	0.183337	959
63658	Soroti	33.61668	1.716681	1132
63702	Mbarara	30.65001	−0.616679	1412
63726	Kabale	29.96669	−1.250005	1867
63680	Kampala	32.61668	0.316673	1144
63705	Entebbe Airport	32.45001	0.050001	1155
63682	Jinja	33.18334	0.450009	1175
63684	Tororo	34.16667	0.683347	1170

**Table 2:** Basic information about the data files is available in the present article. In the table ✓: means same information; **x**: missing variable air pressure at an elevation of the station in some weather stations.

	Weather stations												
Variable	Arua	Soroti	Tororo	Jinja	Kasese	Entebbe Airport	Gulu	Masindi	Mbarara	Kabale	Kampala	Unit	Data period
Date	✓	✓	✓	✓	✓	✓	✓	✓	✓	✓	✓	Date	2020–2022
Air temperature at 2 m above ground level	✓	✓	✓	✓	✓	✓	✓	✓	✓	✓	✓	*o_C_*	2020–2022
Dew point temperature at 2 m above ground level	✓	✓	✓	✓	✓	✓	✓	✓	✓	✓	✓	*0_C_*	2020–2022
Relative humidity	✓	✓	✓	✓	✓	✓	✓	✓	✓	✓	✓	%	2020–2022
Wind direction	✓	✓	✓	✓	✓	✓	✓	✓	✓	✓	✓	direction	2020–2022
Wind speed	✓	✓	✓	✓	✓	✓	✓	✓	✓	✓	✓	km/h	2020–2022
Air pressure at an elevation of the station	✓	✓	✓	✓	✓	✓	✘	✘	✘	✘	✘	hPa	2020–2022
Precipitation	✓	✓	✓	✓	✓	✓	✓	✓	✓	✓	✓	mm	2020–2022
Total cloud cover	✓	✓	✓	✓	✓	✓	✓	✓	✓	✓	✓	oktas	2020–2022
Cloud cover by high-level cloud fraction	✓	✓	✓	✓	✓	✓	✓	✓	✓	✓	✓	oktas	2020–2022
Height of the cloud base	✓	✓	✓	✓	✓	✓	✓	✓	✓	✓	✓	km	2020–2022
Visibility	✓	✓	✓	✓	✓	✓	✓	✓	✓	✓	✓	km	2020–2022

**Table 3:** The first five uncleaned raw weather dataset for Entebbe Airport station.

SNo	Station-ID	Date	TC	TdC	Hr	ddd	ffkmh	POhPa	Precmm	Nt	Nh	HKm	Viskm
0	63705	31/12/2021 21:00	21.3	19.2	88.0	W	18.5	887.2	0.0/3h	6	2.0	0.6	10.0
1	63705	31/12/2021 18:00	21.5	17.7	79.0	W	7.4	886.2	0.0/12h	5	2.0	0.6	10.0
2	63705	31/12/2021 09:00	21.2	19.3	89.0	SW	14.8	886.5	0.0/3h	7	2.0	0.3	10.0
3	63705	31/12/2021 06:00	19.5	18.8	96.0	CAL	0.0	886.7	10.0/24h	7	3.0	0.3	9.0
4	63705	31/12/2021 09:00	25.8	21.2	76.0	WNW	11.1	884.8	0.0/3h	6	2.0	0.6	10.0

Station-ID: Station identifier; TC: air temperature at 2 m above ground level; TDC: dew point temperature at 2 m above ground level; Hr: relative humidity; ddd: wind direction; ffkmh: wind speed; P0hPa: air pressure at an elevation of the station; Precmm: Precipitation; Nt: total cloud cover; Nh: cloud cover by high-level cloud fraction; HKm: the height of cloud base; and Viskm: visibility. Wind direction; CAL: Calm; N: North; NNE: North-North East; NE: North East; ENE: East North East; E: East; ESE: East South East; SE: South East; SSE: South-South East; S: South; SSW: South-South West; SW: South West; WSW: West South West.

**Table 4:** The first five records of the cleaned dataset for Entebbe Airport weather station.

Date	TC	TdC	Hr	ddd	ffkmh	P0hpa	Precmm	Prechrs	Nt	Nh	HKm	Viskm	Prec_rate	Classification
31/12/2021 21:00	21.3	19.2	88.0	13	18.5	887.2	0.0	3	6	2	2	10	0	2
31/12/2021 18:00	21.5	17.7	79.0	13	7.4	886.2	0.0	12	5	2	2	10	0	2
31/12/2021 09:00	21.2	19.3	89.0	11	14.8	886.5	0.0	3	7	2	2	10	0	2
31/12/2021 06:00	19.5	18.8	96.0	0	0.0	886.7	10.0	24	7	3	3	9	0.41667	2
31/12/2021 09:00	25.8	21.2	76.0	14	11.1	884.8	0.0	3	6	2	2	10	0	2

## 4 Experimental Design, Materials and Methods

The climate R-package and Python (Jupyter Notebook 3.9) were used to collect and clean the data in this article. The climate R-package enabled us to get historical weather data in accordance with World Meteorological Organization standards. The software is free to use, user-friendly, and can run on both Windows and Linux systems [1]. Python, on the other hand, was chosen because it is a high-level programming language with codes expressed in human-readable form that is simple to comprehend and use by any coder. Furthermore, with far too many libraries and functions for statistical and numerical analysis, Python source code is freely available to anyone [2],^3^.

## 5 Data Preparation

This is the process of transforming raw data into a suitable format for further processing and analysis [2]. To accomplish this, the collected data were cleaned to remove inconsistent data formats, duplicates, and errors. During the data cleaning process, we were able to match the data records, create new features and labels, and identify data inaccuracies, resulting in an improvement in data quality. This process involved the following steps:

­ The missing data in each of the variables were identified and counted as true and the non-missing as false with their respective data types.
­ The record /tr for trace in the precipitation variable was replaced with a value of 0.01 [3]
­ Prechrs, Prec_rate, and classification were new features introduced to define precipitation in hours, precipitation amount per hour, and precipitation classification intensity per hour categories respectively.
­ In the variable prechrs the following records 2 h, 3 h, 6 h, 8 h, 12 h, 18 h, and 24 h from precmm were added as records in the Prechrs feature. Thus, we dropped the h symbol to remain with numeric values of 2, 3, 6, 8, 12,18, and 24. Therefore, /2 h, /3 h, /6 h, /8 h, /12 h, /18 h, and /24 h in the Precmm features were also replaced with a space to remain with numeric values.
­ The categorical records in variable ddd: CAL, N, NNE, NE, ENE, E, ESE, SE, SSE, S, SSW, SW, WSW, W, WNW, NW, and NNW were substituted with 0, 1, 2, 3, 4, 5, 6, 7, 8, 9, 10, 11, 12, 13, 14, 15, and 16 respectively. Also, the missing values in the ddd records were replaced by the mode value. The variable ddd: Wind direction indicates where the winds are blowing from.
­ Prec_rate feature which was created, classified as slight (when the precipitation rate is < 2.5 mm per hour), Moderate Rain (when the precipitation rate is between 2.5–7.6 mm or 10 mm per hour), Heavy Rain (when the precipitation rate is > 7.6 mm per hour or between 10 mm and 50 mm per hour), Violent Rain (when the precipitation rate is > 50 mm per hour [4]. Subsequently, the categorical values in Prec_rate features were converted to numeric values by assigning: Slight, moderate, Heavy, and Violent values 2, 1, 0, and 3 respectively.
­ The numeric and float data type variables are TC, TdC, Hr, P0hPa, Precmm, Nt, Hkm, and Viskm. Also, the categorical ones are ddd, Prechrs, Prec_rate, and classification.
­ The true numeric values or float were replaced by their average values and the categorical records were replaced by the mode.
­ The dates were arranged in the form of time stamps ready for analysis and visualization in Python.
­ Data types of every feature were changed to integers and floats.
­ The variables that had above 50 percent of missing data were not considered for this experiment [[5], [6], [7]].

Following data preparation, the cleaned and analysed dataset is provided in Table 4. The table below shows the first five recordings acquired from the Python programming environment for the same weather station Entebbe Airport. The complete datasets for all 11 weather stations can be found in the Harvard Dataverse repository via the URL provided.

**Fig. 3:**
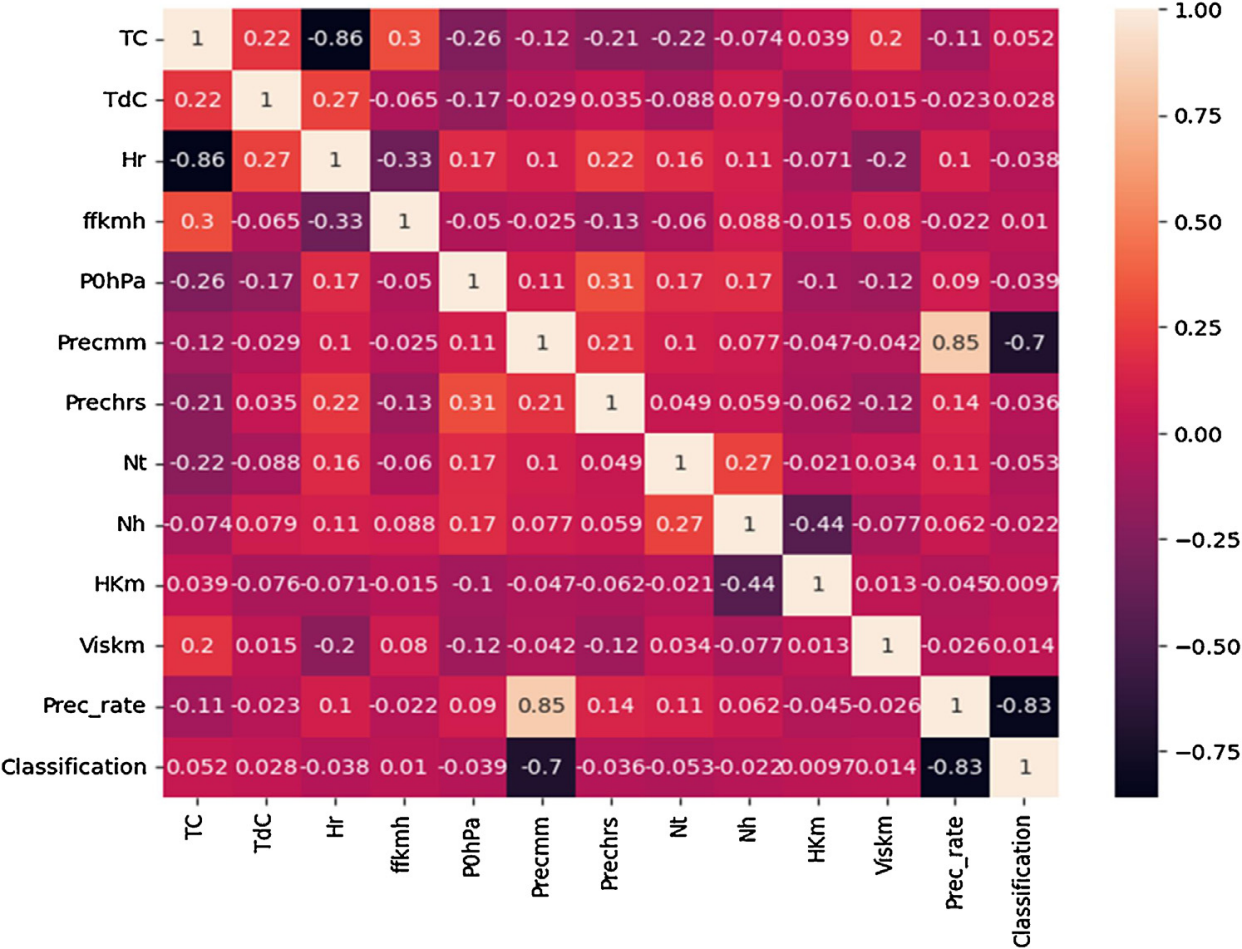
Shows how independent variables are correlated with Precmm and Prec_rate.

Using the dataset represented by Table 4 of the size 2927 records and 15 fields, experiments were conducted on the Entebbe and Masindi weather stations dataset using statistical regression algorithms such as simple linear regression (SLR), polynomial regression, and multiple linear regression (MLR). However, these could not be used in the prediction of Precipitation and Precipitation rate per hour because none of the independent variables were strongly or moderately correlated to the dependant variable as shown in Fig. 3.

**Fig. 4:**
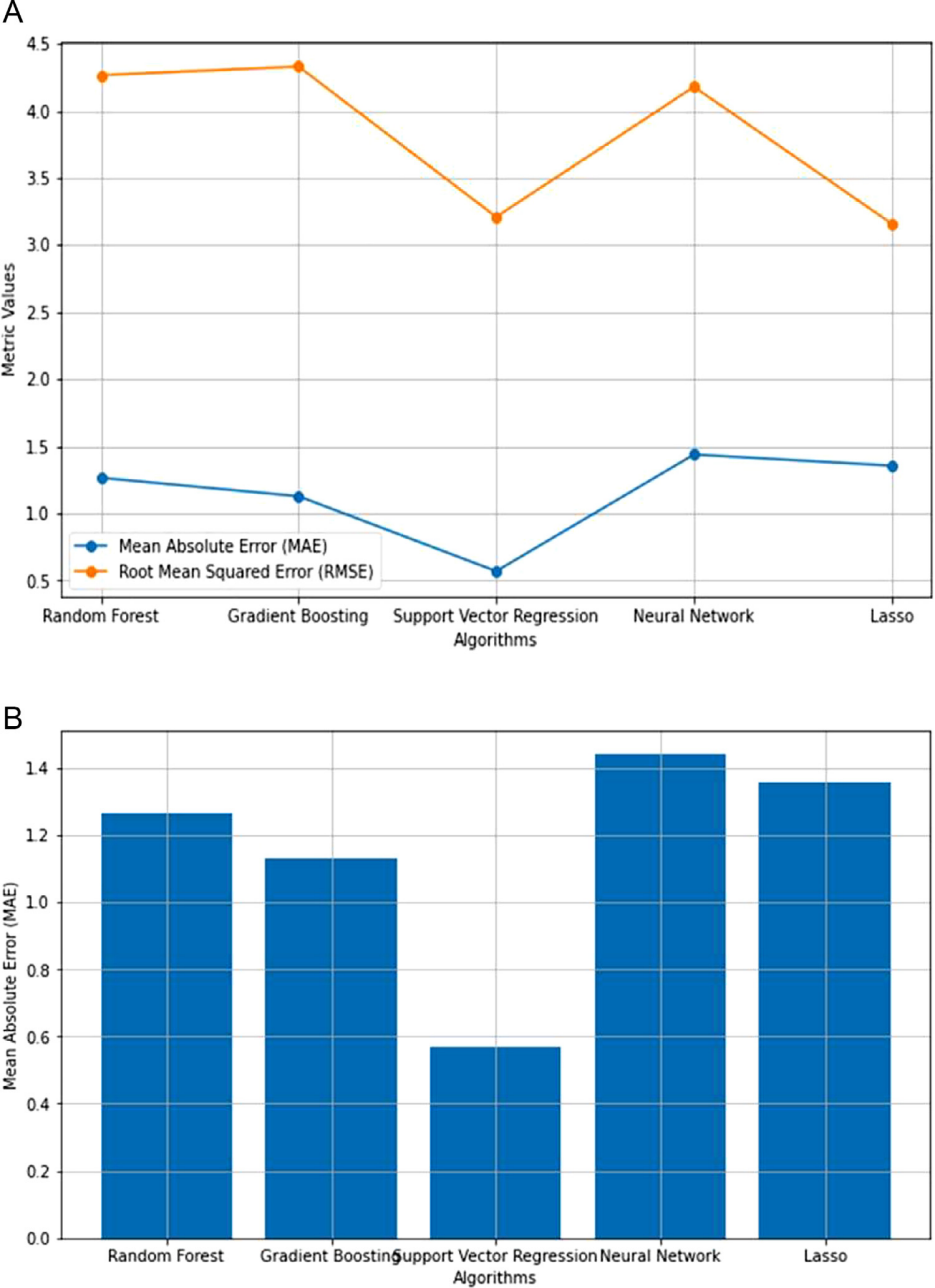

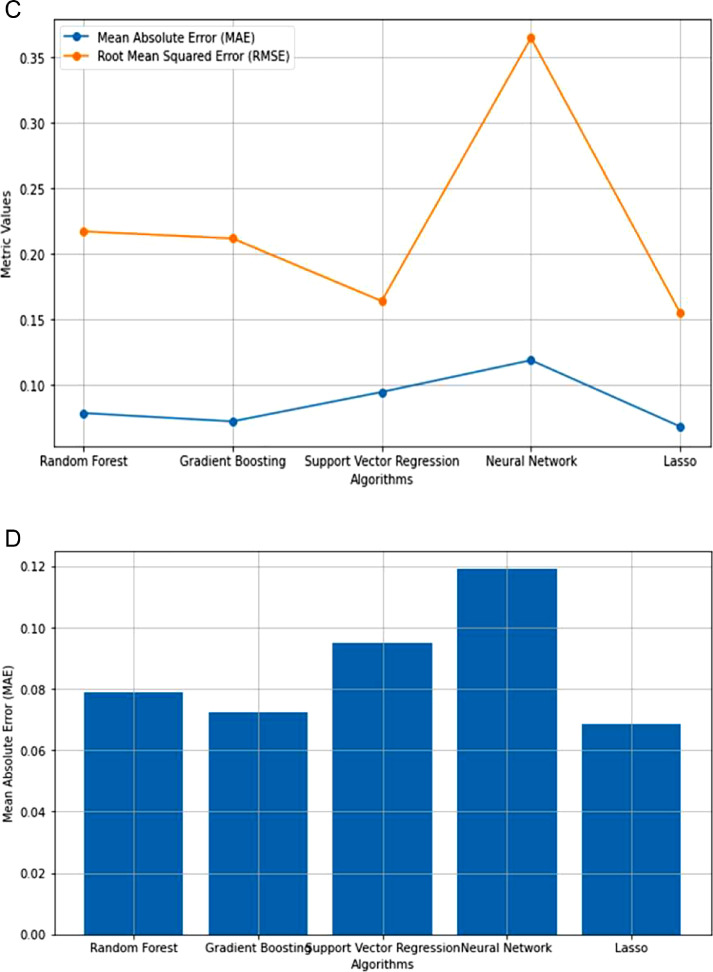
**(a):** Comparison of regression algorithm performances in predicting Precmm at Entebbe. **(b):** Mean Absolute Error across regression algorithms in Precmm Prediction at Entebbe. **(c):** Comparison of regression algorithm performances in Prec_rate Prediction at Entebbe. **(d**): Mean Absolute Error across regression algorithms in Prec_rate prediction at Entebbe.

**Fig. 5:**
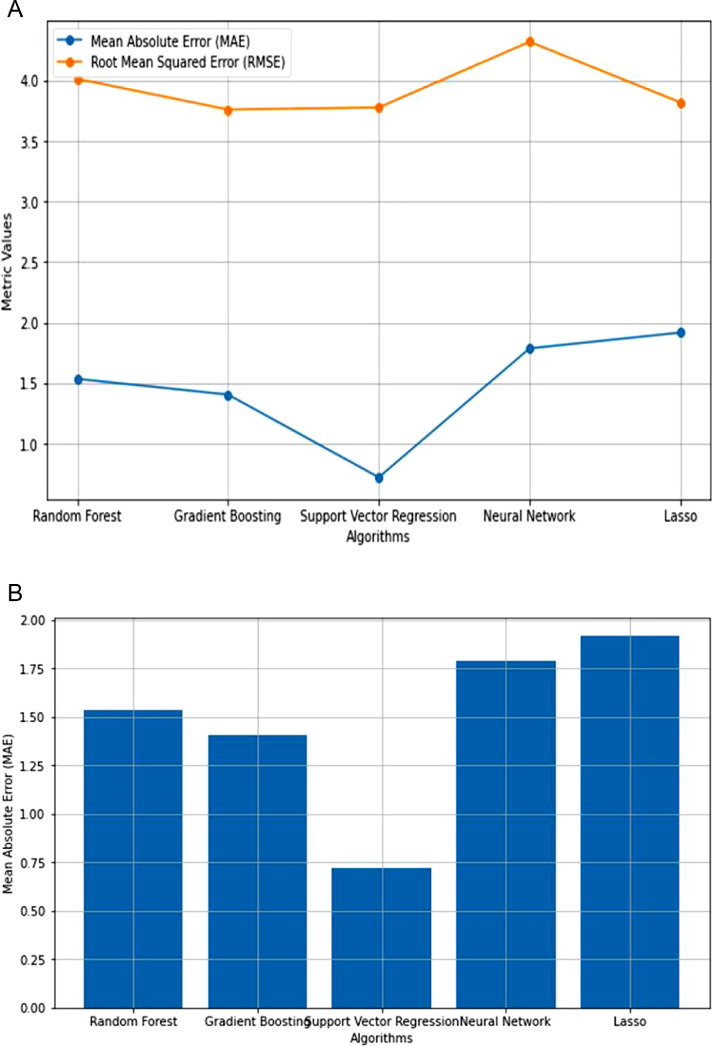

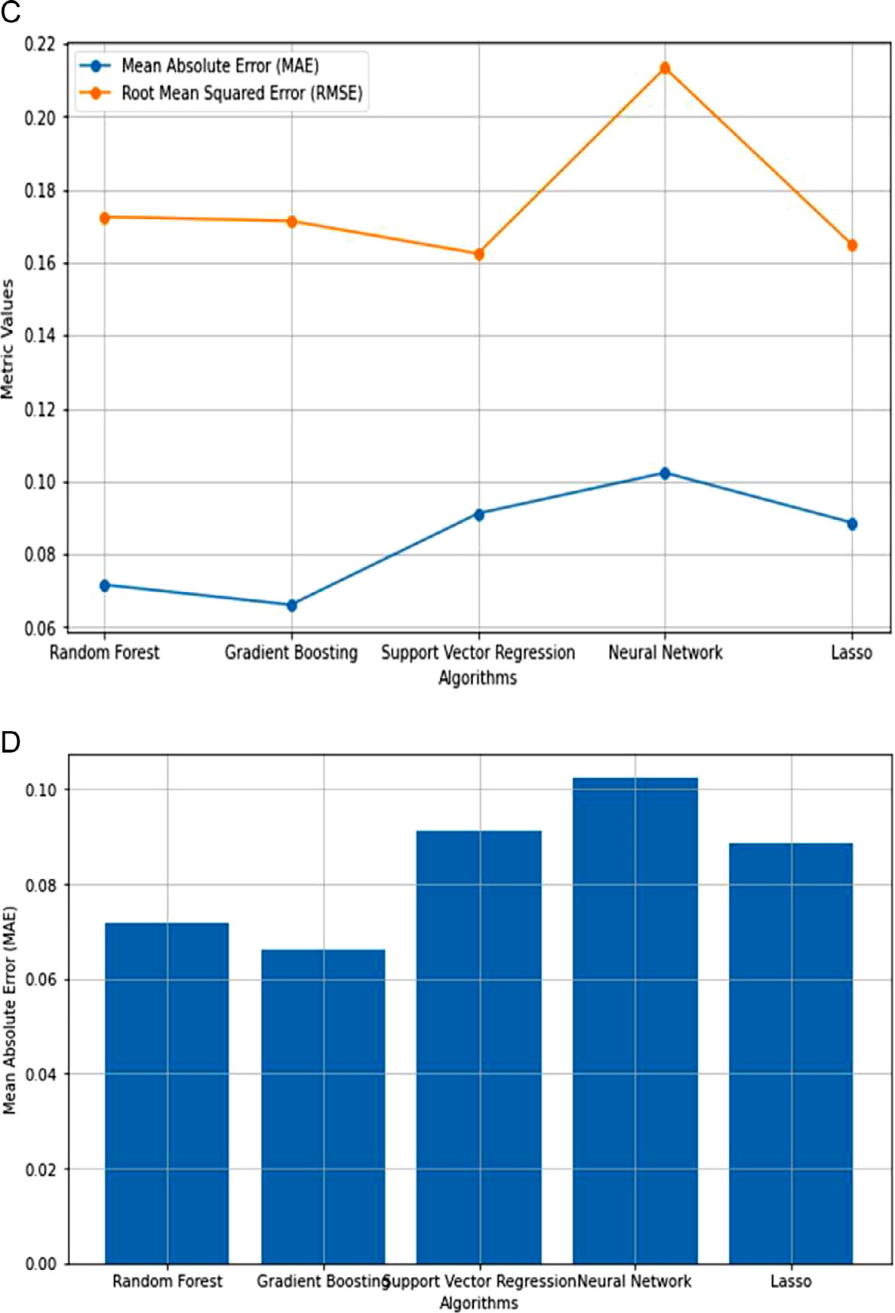
**(a):** Comparison of regression algorithm performance in Precmm prediction at Masindi. **(b):** Mean Absolute Error across regression algorithms in Precmm prediction at Masindi. **(c):** Comparison of regression algorithm performances in Prec_rate prediction at Masindi. **(d):** Mean Absolute Error across regression algorithm in Prec_rate prediction at Masindi.

Because of the lack of strong correlation between the parameters, as shown in Fig. 3, machine learning regression approaches are the best for prediction using the features proposed in this article. Following that, machine learning algorithms such as Random Forest, Gradient Boosting, Support Vector, Neural Network, and Lasso regression were used to predict precipitation and precipitation rate per hour. The performance of the machine learning algorithms was compared, as shown in Table 5 and Fig. 4, Fig. 5.

**Table 5:** The performance of different machine learning regression algorithms for Entebbe and Masindi weather stations.

Station		Random Forest Regression		Gradient Boosting Regression		Support Vector Regression		Neural Network Regression		Lasso Regression	
		MAE	RMSE	MAE	RMSE	MAE	RMSE	MAE	RMSE	MAE	RMSE
Entebbe63602	Precmm	1.2657	4.2654	1.1284	4.3304	0.5697	3.2094	1.5207	4.2137	1.3565	3.1596
	Prec_rate	0.0789	0.2173	0.0725	0.2119	0.0949	0.1624	0.1328	0.3345	0.1526	0.2774
Masindi63654	Precmm	1.5349	4.0133	1.4047	3.7597	0.7212	3.7769	1.5629	4.5251	1.9170	3.8190
	Prec_rate	0.0717	0.1725	0.0662	0.1714	0.0912	0.0912	0.1100	0.2209	0.886	0.1648

The Mean Absolute Error (MAE) and Root Mean Squared Error (RMSE) are known prediction error metrics [8]. However, Mean Absolute Error is considered the most accurate error metric [8]. The results in Figs. 4 and 5 show that the MAE error results are lower than the RMSE implying that our prediction is reliable for the case of justifying the validity of our new dataset.

## Ethics statement

The study does not involve experiments on humans or animals.

## CRediT authorship contribution statement

**Andrew Gahwera Tumusiime:** Conceptualization, Data curation, Methodology, Writing – original draft. **Odongo Steven Eyobu:** Supervision, Data curation, Writing – review & editing. **Isaac Mugume:** Supervision, Investigation, Writing – review & editing. **Tonny J. Oyana:** Writing – review & editing.

## Data Availability

Cleaned Weather Dataset for Uganda (Original data) (Dataverse)
